# Some Remarks on Cataract

**Published:** 1858-01

**Authors:** C. S. Fenner

**Affiliations:** Memphis, Tennessee


ART. I.—*Some Remarks on Cataract.* By C. S. FENNER, M.D., of Memphis, Tennessee.

I AM not aware that we have any statistical records going to show that cataract is more common in one country or section of country than another, or that any particular class of persons, sex, color, or those following any specified occupations, are peculiarly liable to it. Mr. Mackenzie remarks,—"In the early years of my practice I met with a greater number of stocking-weavers affected with cataract than of any other single trade." In the later editions of his work, he does not say that from more extended observations he has found the unusual prevalence of cataract among this class of laborers to continue; hence we may conclude that the greater number of cases occurring among stocking-weavers for a limited time was accidental. Cataract is frequently met with in the Southwest. I have never seen a case other than traumatic that I could with certainty attribute to any particular cause. I believe it to be the result of defective nutrition, independent of inflammatory action. Capsular cataract I regard as always the result of inflammation. The disease is evidently hereditary: according to my observations the mother is more apt to transmit the tendency than the father, and to her daughters rather than to her sons. To distinguish cataract from incipient amaurosis, the catoptrical test is undoubtedly the best, and should always be resorted to when there is the least doubt in regard to the nature of the disease.

*Prognosis.*—It is a very common opinion with the public at large, and prevails to a considerable extent among the medical profession, that cataract is a disease of but little importance; that by a slight operation the obstruction to vision can readily be removed and sight restored, estimating too lightly the danger of an operation for the removal of cataract upon so delicate an organ, from changes produced by subsequent inflammation. It is always best to assure patients with cataract that the result of efforts for relief cannot with certainty be foretold; that according to the experience of the most skilful surgeons, not more than from one-half to three-fourths of those operated on recover useful vision; and sometimes the successful results are even less favorable. Dr. Tartra says,—"It is generally thought that two out of five patients operated on for cataract recover their sight." M. A. Saez, of the General Hospital of Madrid, reports 525 operations for cataract, of which 84 failed. Of the 441 cases reported as successful, I suppose he includes all in which vision was improved. His statistics would have been more satisfactory had he stated the number of entirely successful cases, and the number in which the improvement of vision was but partial.

Professor Roux reports 115 patients operated on for cataract, of whom 73 recovered their sight. Daviel "operated on 34 cases; 17 of which were perfectly restored to sight, 8 saw indifferently, and 9 received no benefit." M. Sichel furnishes, during a period of nine years, a record of 1026 operations on 641 patients; of which 783 succeeded, 114 were partially successful, and 117 failed entirely.

If patients with cataract are apprised beforehand of the dangers attending an operation, and the consequent uncertainty of the final result, they will be better prepared to submit to disappointment in case of failure, and will be less disposed to reflect on the operator or attribute to him a want of skill. On this subject Mr. Mackenzie's remarks are very appropriate. He says,—"With regard to the prognosis, inclusive of the probabilities of cure, practitioners are too much in the way of raising sanguine hopes in the minds of patients affected with cataract, that by surgical operations on the eyes their sight may be almost perfectly restored, not weighing with sufficient consideration the frequency with which other morbid changes in the organ of vision are associated with this disease, especially in advanced life, such as dissolution of the vitreous humor and imperfect sensibility of the retina. The dangers, too, attending the operations for cataract are much too lightly estimated in pronouncing an ultimate prognosis. The risk of a badly performed operation, and that of disorganization from inflammation and other causes, after even the best-performed one, are too much left out of view." He further adds,—"In the practice of one thoroughly acquainted with eye-diseases, able to discriminate the cases fitted for extraction and those fitted for division, able to perform those operations well, and careful and skilful in the after-treatment, I should think three-fourths of those operations would recover useful vision, two-thirds excellent vision."

Patients with cataract often express considerable surprise when told that there is any doubt in regard to the final result of an operation, and that it is necessary for them to submit to a rigid course of treatment to allay subsequent inflammation and give them the best possible chance for recovery. While penning these pages, an elderly lady, with double cataract, arrived here on the cars on Friday, called at my office the same evening, wished an operation performed the next morning, and had made arrangements to return home on Monday. I declined attending her case unless she would remain at least a month, and longer if necessary. She went home, promising to return in a short time prepared to stay as long as the necessities of her case required.

I have occasionally succeeded in making a perfectly clear pupil, with but little improvement in vision, although previous to the operation the perception of light and the mobility of the iris seemed to indicate considerable activity of the retina. With me the chances of success have diminished after the age of fifty, while a large proportion of operations on persons under that age have been entirely successful. After the age of sixty-five or seventy, I think the prospects of a cure more favorable than in middle-life. Females of advanced age, according to my experience, do not bear operations for cataract as well as males, being much more liable to subsequent chronic internal inflammation, aggravated by atmospheric influences.

*Medical treatment of cataract.*—Occasionally our journals contain rereports of cases of cataract cured by medical treatment alone. Most of such reports are from men who have not had large experience in eye-diseases, and a reasonable inference may be drawn that they have been mistaken in regard to the true nature of the disease. If the history of such cases has been given, we find the opacity generally preceded by inflammation, and is most likely caused by an effusion of lymph in the pupil, covering the entire capsule, producing spurious cataract. If true lenticular cataract ever disappears from the result of medical treatment, it seems strange that cases of the kind should not occasionally come within the notice of such men as Beer, Leport, Denonvilliers, Gosselin, Mackenzie, Lawrence, Dalrymple, Sichel, Desmarres, Hays, Gross, and many other eminent surgeons, whose professional standing and extended experience would seem to preclude the possibility of an erroneous diagnosis.

*Seasons of the year best adapted to operations for cataract.*—Most English and American surgeons pay but little attention to the choice of seasons in their operations for cataract, while the Germans and French regard it as a matter of primary importance. In this latitude, I am satisfied that some seasons are much more favorable for all severe operations on the eye than others, and when I can conveniently control the time, I would select the months in the following order: September, October, November, May, June, and the first two winter months. February, March, and April are peculiarly unfavorable in this climate, owing to the frequent atmospheric changes, and the great tendency to rheumatic affections during these months greatly increasing the risk of inflammation. Steady, dry, cold weather is not particularly unfavorable, but we rarely have that condition of the atmosphere for any considerable length of time. Mr. Tyrrell says,—"I am perfectly satisfied that the operation of cataract is most hazardous during the cold and damp seasons; so much so, that it is only under very peculiar circumstances that I ever operate in the period from October to March. The proportion of unsuccessful cases has been very much lessened since I have discontinued to operate in cold and damp periods of the year." Dr. Schlagintweit, of Munich, an old pupil of Beer, remarks,—"If the weather were mild in the months of October and November, I would not decline to operate; but in frosty, or in keen or variable cold weather, or in the heats of summer, I would never think of it." Dr. Gulz, of Vienna, says,—"We have no formal or positive rule not to operate at any period of the year. But then, as a matter of fact, we do not like to do it during the unfavorable and trying seasons of the year, nor, in fact, do we then operate much. Good and steady weather, with an agreeable temperature, is what we desiderate; and everything that interferes with this—the cold and snowy weather of winter, the piercing and chilly blasts of spring, or the very hot season, or a very wet one—we try as much as possible to avoid; and hence May and July, September and October, are the months we prefer."

*Should an operation be performed when there is cataract in one eye only, vision in the other being perfect?* In young persons with single cataract, I usually advise an operation, if the disease is uncomplicated and the general health and constitution of the patient good. Cataract in one eye is a considerable blemish, and is very annoying, particularly to females. They are unable to see objects on that side distinctly; they have to turn the head toward the shoulder in conversing with persons, or viewing objects on the blind side, which gives them an awkward air; besides, they are more liable to injuries. Severe inflammation rarely follows operations for cataract on healthy young persons, consequently the risk of destructive inflammation extending to the sound eye is too small to be calculated on. The objection often made to an operation, that the difference in the focal distance of the two eyes, after the lens in one has been removed, will give rise to double vision, I have found not to hold good. Patients on whom I have operated in such condition have uniformly expressed themselves as gratified with the result: the deformity has been removed, and the scope of vision extended. In young persons the eye, after the removal of the lens, gradually accommodates itself to the loss, and patients often have very good and distinct vision without the aid of cataract-glasses: the younger the person the more perfect the sight becomes without magnifiers. Very few persons have the two eyes of precisely the same power, yet very few have double vision. Mr. Mackenzie says,—"It is rarely the case that the two eyes, even in the same person, correspond in refractive power.

The left, partaking, perhaps, in the tendency to weakness and disease which so frequently attaches itself to the left side of the body, is often found to be short-sighted. Few are aware of the disparity which often exists between their eyes, until some accidental circumstance leads them to make a comparative trial of the two; and it is by no means uncommon to meet with individuals who, on making the experiment, have discovered that one eye was greatly defective, or even entirely blind." If the two eyes of the same person rarely correspond in refractive power, and yet vision be good, surely the fear of diplopia should not deter one from the removal of an opaque lens. The improvement in the scope of vision, the removal of a serious blemish, and possibly the prevention of the disease from extending to the sound eye from sympathetic action, and the small risk of destructive inflammation, induces me to advise an operation in such cases. In young persons a cataract often exists in one eye for many years without extending to the other, while in middle and advanced age if one eye becomes cataractous, the other rarely remains good more than two or three years, and sometimes not as many months.

*Surgical operations for cataract.*—Extraction, displacement, and division are the three operations performed for the removal of cataract, and each method is modified according to the pecularities of the case, or the views and skill of the surgeon. When the lens is soft, as is generally the case in young persons, division is the most appropriate operation. A free opening should be made in the central portion of the anterior capsule, and the needle repeatedly passed through the soft lens without displacing it from its natural position. Care should be taken to make the capsular opening free and leave no part floating in the pupil. This is sometimes difficult, as the membrane is transparent, very elastic, yields before the needle, and although a large opening may be made, the fragments, retaining an attachment, rise up, float in the pupil, inflame, become thickened, opaque, and thus give rise to secondary capsular cataract.

M. Malgaigne, in 1842, advanced the opinion that the true capsule never lost its transparency, but that the opaque matter was deposited on its lenticular surface. This opinion has been controverted, and has given rise to much discussion, in which Stellwag, Sichel, Gnepin, Szokalski, Haring, Bosck, and A. Richard have taken an active part. Stellwag has microscopically examined about fifty capsular cataracts, and in every instance he states that the result confirms the opinion first advanced by Malgaigne. I have always found that in traumatic capsular cataract the membrane is much thicker than natural, tough, and its elasticity gone or very much diminished; the anterior surface has always retained its natural polish, while its posterior side is rough; an evidence that the thickening is caused by a deposit on the lenticular surface of the membrane. In couching, the anterior capsule is usually displaced and carried down with the lens; in extraction, the passage of the lens through the opening made in the capsule generally destroys the membrane to its circumference. Secondary capsular cataract will occasionally occur after each of the operations for the removal of the lens. I have seen the edges of large rents in the anterior capsule come together and unite, forming a dense white cicatrix, with opacity and thickening of the membrane, so as almost entirely to destroy vision.

*Displacement.*—When the cataract is hard, couching is the operation generally preferred by American surgeons, although by most English and German authors it is placed lowest in the scale. My own experience in displacement has not been so favorable as that reported by some American operators. Dr. Hewson, in his edition of Mackenzie, quotes from Dr. Pancoast, stating that he has operated by lateral displacement "in about twenty cases of hard cataract, without any permanent failure." Dr. Nott, of Mobile, in the *New Orleans Medical and Surgical Journal*, vol. xii., No. 2, gives a flattering account of the result of his operations for cataract by displacement and solution. He says, "I have now been practicing in Mobile nearly twenty years, and during this time have had occasion to operate very frequently for cataract,—very few cases of which have been congenital and very few under adult age,—and though my statistics have not been carefully kept, I am satisfied that four out of five of the cases operated on have been soft, and not a few milky. To this fact I attribute a degree of success in my operations which is remarkable, for I do not pretend to any peculiar merit as an oculist. I have always commenced my operations upon the presumption that the cataract was to be a hard one, and if so, my plan is to displace it in the usual way. I generally find the lens too soft to bear the pressure of the needle, and have broken it up and left it to be absorbed *in situ*. I really approach a case of cataract with more confidence of success than any capital operation in surgery. The question arises, is there anything in our climate or other local circumstances which can explain this peculiarity of our cataracts, or is it chance which has thrown so large a portion in my way?" My experience differs somewhat from Dr. Nott, in reference to the relative frequency of soft and hard cataracts. I have repeatedly had occasion to operate on cases of this disease, from the eastern and southeastern parts of Mississippi and the contiguous counties of Alabama, and I can now call to mind but few cases of soft cataract. Semi-soft I have found more common than any other form; either the entire lens, or the central part having sufficient firmness to be moved before the needle when that instrument has been used. Dr. Nott is well known to the profession as one of our ablest surgeons, and doubtless uses the needle with great skill; but I am inclined to think his degree of success must be attributed to two causes, namely, first, the great number of soft cataracts chance seems to have thrown in his way, the removal of which by solution is rarely followed by destructive inflammation; and, secondly, to the fine climate, pure air, and equable temperature of Mobile during the winter and spring months, the period of year that patients from the country, tributary to Southern seaport cities, usually leave home for surgical operations. That a cold, damp, and changeable atmosphere is prejudicial to patients who have been operated on for cataract, is proved by statistics furnished by M. A. Saez, of Madrid, who states that in the spring of 1842 he had fifty-three cases under treatment; thirty-seven were in a dry, warm, and comfortable ward, and sixteen in one that was cold and damp. Of those in the first-named ward, thirty-one were cured; in the latter, only eight. In my operations for cataract by displacement, I have often had to combat a subacute chronic internal inflammation, sometimes coming on several weeks after the operation, and seemingly induced by atmospheric influences. The presence of a hard lens in the vitreous humor in some instances causes irritation, followed by slight redness of the conjunctiva, inability to bear light, increased secretion of tears, a sensation of fulness in the eye, soreness to the touch, and pain at times extending to the terminal branches of the fifth pair of nerves. Iritis may supervene with effusion of lymph in the pupil, but generally the pupil remains clear, the vision gradually fails, until the eye becomes almost amaurotic. I have had this result to occur most frequently in delicate, aged females. Mr. Lawrence remarks that "internal inflammation commencing in the iris, and proceeding slowly, is a common occurrence after depression;" and Mr. Mackenzie, that "chronic inflammation of the internal textures of the eye is a frequent consequence of depression or reclination. It is not attended by much pain, but prevents the eye from ever attaining a degree of healthiness sufficient to render it useful. The patient, perhaps, retains a considerable degree of recovered sight for some weeks after the operation; but epiphora, varicose dilatation of the external blood-vessels of the eye, and in general a contracted, but sometimes a dilated pupil supervening, the sight becomes weak, and in a few months is extinguished." I have repeatedly had cataract patients return to their homes able to see to read fine print with ease, yet in a few months scarcely able to walk about, and I have witnessed the same final termination in those who have been operated on by experienced surgeons of the North and East; they returned home with good vision, but this gradually failed them, until they became amaurotic. Such being the occasional result, no case of cataract should be reported as cured, unless the state of vision is known six or twelve months after an operation; and if operators would keep their patients in view for that length of time, the final result of their operations, I

am inclined to think, would not be so favorable as they sometimes report.

*Displacement through the cornea.*—In the early years of my practice I performed some nine or ten operations for cataract by displacement, by introducing the needle through the cornea, believing a wound of that part of the eye to be less likely to induce inflammation than a similar one through the sclerotica; but I have entirely abandoned that mode of operating. The point of the needle does not have sufficient play; the lens cannot in every instance be carried sufficiently deep into the vitreous humor to break up its attachments, particularly at its lower edge, so that if pushed down it is apt to rise again; the anterior capsule cannot be lacerated with the same ease as in the operation through the sclerotica, and it is very difficult to remove shreds of capsule remaining in the pupil.

*Extraction.*—To remove a hard opaque lens entirely from the eye is undoubtedly the most perfect, and, when it can readily be accomplished, the preferable operation, as it does not interfere with the structure of the posterior chamber; but the delicacy of the operation, and the amount of experience and mechanical tact required to perform it well has induced many operators to resort to displacement, which is much more simple, and, in inexperienced hands, the safest operation. I believe that when extraction is skilfully performed the patient has a much better chance of recovering and retaining his sight than after depression. Mr. Hamilton, of Edinburgh, from information derived during a tour of observation through Germany, says,—"It is regarded almost as an axiom at the Vienna school, that if the case be well selected, and the operation be adroitly performed, extraction is, beyond all comparison, the most satisfactory procedure for all concerned. At the same time it is maintained that it requires considerable skill and practice, so that when individuals are so placed that they have not every advantage, they are then prone to look about for arguments against it, and for reasons why other methods should be preferred, which, in reality, amounts to the admission that extraction is too difficult for them. Hence the rarity of good extractors, and these being almost confined to capitals and other large cities, and to few even in them; hence so many preferring other operations—seldom or never having recourse to this one; hence, also, the many objections brought against this operation, which those who are skilful in its performance know nothing about, finding it truly successful and free from injurious consequences." Mr. Mackenzie adds:—"That extraction is the proper mode of removing a hard cataract is an assertion of the truth of which those who have had any considerable experience in the treatment of eye-diseases appear to be as firmly convinced of as they are that soft cataract may safely and satisfactorily be cured by division." According to my experience, extraction is generally followed by more immediate inflammation than occurs after the operation of depression, but it can usually be controlled by rapid depletion, and after it has subsided the patient is less liable to subsequent subacute posterior internal inflammation, often permanently injuring vision. I have occasionally extracted in one eye and displaced in the other, and in such instances the former operation has generally given the most permanent and satisfactory vision. Disorganizing inflammation following displacement is attended by excruciatingly severe pain, resulting from an increased secretion of aqueous humor, the formation of pus, or an effusion of lymph in the interior of the eye, distending its unyielding coats. In one instance I found it necessary to open the cornea to relieve the internal pressure. Inflammation following extraction is much less painful, as the opening in the cornea allows the escape of the redundant fluids, relieving the eye from the pain of excessive distension. In a prominent eye I prefer making the superior section of the cornea, which can be done with equal facility as the inferior; the constant pressure of the upper lid, and its levator muscle, has a tendency to keep the cut surfaces in contact, thus favoring union by the first intention. If the inferior section is made, and the wound does not speedily unite, in opening the eye the lower lid is apt to become entangled in the wound, and interfere with union; in such cases there is swelling of the conjunctiva at the edge of the cornea, which, in moving the eye, is irritated by frequent contact with the margin of the lower lid. When a skilful assistant is not at hand, rather than trust to an awkward or inexperienced one, I prefer to operate without assistance, holding the lid and steadying the eye with the fingers of one hand and using the knife or needle with the other, alternating the hands according as the right or left eye is to be operated on. If, however, the eye is deep-seated in the orbit, the brow projecting, the opening of the lids small, and the orbicularis palpebrarum active, an assistant cannot well be dispensed with in making the incision in the cornea; indeed, in such cases it is scarcely advisable to attempt extraction.

*Treatment of secondary capsular cataract.*—This affection is common after the removal of the lens. I have had it occur after my own operations, and have frequently met with it in cases operated on by others. Fragments of capsule retaining an attachment, so that the circulation can be kept up, are never absorbed, but inflame, become thickened and opaque, and the free edges float in the pupil and obstruct vision. I formerly attempted to remove this membrane by means of a needle introduced through the sclerotica, but generally without success; as it is very firm and tough, and, floating in the aqueous humor, it yields before the needle, so that it is impossible to penetrate or entangle the point in such a manner as to tear the capsule from its attachment; and if carried into the vitreous humor out of the axis of vision, on the withdrawal of the instrument it floats back into the pupil again, and the patient is not benefited by the effort. The most effectual way of restoring vision in such cases is to open the cornea at its temporal margin, introduce a pair of delicate forceps, seize the membrane and withdraw it from the eye. The canula-forceps, recently invented by M. Charrière, are admirably adapted to the removal of secondary capsular cataract, requiring but a slight corneal opening to give access to the opaque membrane. Occasionally we find the capsule adherent to the posterior surface of the iris. The following case came under my charge in the spring of 1854:—A negro woman had been operated on for cataract some years previously, in Charleston, S.C. When I saw her the right eye was staphylomatous; the pupil of the left was entirely covered with a dense, firm, white membrane, attached to the uvea, so that the iris was motionless and the pupil rather contracted. She had some perception of light, and could tell the situation of the windows, and distinguish the shade of the hand passed before the eye, when exposed to a strong light. In the presence of the late Professor Wooton, and Drs. Harris and Smith, I opened the cornea at its temporal margin, and with a straight cataract-needle, its point delicately held against the centre of the thickened capsule, I drilled a hole through it by rotating the instrument between the thumb and finger. I then bent the point of an iris-hook at right angles, introduced it through the central opening, and entangled the point in the posterior part of the membrane, near the pupillary margin, and by gentle manipulation succeeded in detaching it from the iris at its superior and nasal edge. I then withdrew it through the corneal opening until that part of the iris retaining an attachment was everted, when I dissected it loose with a delicate knife, and removed it entirely from the eye. The iris was not wounded, and immediately returned to its natural position, leaving a perfectly clear, black, but motionless pupil. The incision in the cornea healed in forty-eight hours, without pain, and scarcely perceptible conjunctival redness. Vision was improved so as to enable the woman to find her way about, but not sufficient to see small objects distinctly.

*In double cataract ought both eyes to be operated on at the same time?*—As a general rule, I think it best to perform a double operation, and I see no reason to depart from this procedure, if extraction is deemed to be the preferable operation; but if the lens removed is found to be hard at its centre, with soft periphery, I prefer to use the needle on the second eye, lacerate the capsule, and allow the soft part to be dissolved, and extract the nucleus at a subsequent time through a small section of the cornea; indeed I consider this mode of operating the best, whenever this state of the lens is previously known to exist; and if any part of the capsule remains in the pupil, it can be removed with the hard nucleus. I have noticed that patients with double cataract, after one eye has been operated on, have great dread of an operation on the other, even if the restoration of the first eye was complete, and attended with but little pain. If the attempt on the first eye fail from suppurative inflammation, or other cause, and is attended with severe pain, patients often refuse to submit to a second operation, believing it would be unavailing, subject them to unnecessary suffering, and, perhaps, deprive them of even the perception of light. Cataract patients generally come from a considerable distance, are not unfrequently in indigent circumstances, and consequently find it inconvenient to return at a subsequent period for a second operation. In nearly every variety of ophthalmia, when both eyes are affected, one is always worse than the other—alternating, perhaps, the acute symptoms yielding in one while they increase in the other; and I have noticed the same alternation of acute inflammation in the two eyes that had been operated on for cataract at the same time, one inflaming severely, while the other is scarcely sore at all. In May last I performed a double operation on an old man for soft lenticular cataracts with small hard central nuclei. I lacerated the capsules, broke up the lenses, and displaced the central portions at one sitting. The right eye inflamed considerably, was painful, red, and tender to the touch, and could not bear the light. The left one did not inflame at all, and gave him no uneasiness whatever, until at the end of three weeks, when, during one night the pain and tenderness suddenly changed to the left eye, and the right became apparently well. The acute symptoms soon yielded; a portion of opaque capsule remaining in the pupil of the left eye, I removed it through an opening in the cornea, and the patient went home two weeks after the last operation, able to read small print with the assistance of magnifiers.

It is not unfrequently the case that the first operation succeeds, and one performed at a subsequent time on the other eye fails, and *vice versa;* and sometimes both eyes are lost when operated on at different periods. That these results occur more frequently after a double operation, I very much doubt. Statistics from the records of surgeons of large experience would be very useful on this point, and would definitely settle the question. Dr. Sloan, house-surgeon to the Leicester Infirmary, has recently published the result of two cases of double extraction, and adds:—"I attach great value to any statement of Mackenzie's regarding ophthalmic surgery; but I believe, and I hope to be able to prove to you, that his opinions concerning double extraction are erroneous. Every one who has seen many cases of diseases of the eyes, must be aware of the great sympathy that exists between them; and I was taught to consider the extraction of both lenses at one sitting a most hazardous proceeding, inasmuch as if inflammation came on in one eye, the other would most probably become affected by sympathy, and that it was therefore advisable to allow some months to elapse between each operation. * * * * I learned from Mr. Paget that his father, whom he succeeded in the office of surgeon to the Infirmary, always performed double extraction; that he himself has always followed the same plan; and their combined experience extends over a period of sixty years. I have been told that his late colleague, Mr. Nedham, did, and I know that his present colleagues, Messrs. Macaulay and Beufield, do extract cataractous lenses by the double operation. These gentlemen are convinced that the greater risk of inflammation after double extraction is a myth—a plausible hypothesis not founded on facts; and the truth of which, I believe, has not been previously questioned in this country." The remarks of Dr. Sloan are not only sensible, but the views he so ably advocates have stood the test of experience.

